# Evaluation of Adaptive Changes by Non-Invasive Imaging in Hepatic Vein Outflow Obstruction

**DOI:** 10.1155/1995/79058

**Published:** 1995

**Authors:** P. Gertsch, J.-N. Vauthey, C. Looser, J. Triller, L. H. Blumgart

**Affiliations:** 1 Clinic of Visceral and Transplantation Surgery Inselspital Bern University of Bern Bern 3010 Switzerland; 2 Department of Radiology Inselspital Bern University of Bern Bern 3010 Switzerland; 3 Dept. of Surgery University of Hong Kong Queen Mary Hospital Pokfulam Road Hong Kong

## Abstract

Hepatic vein outflow obstruction induces remarkable changes of intra–hepatic blood circulation; the significance of these changes remains uncertain. Six patients with obstruction of the hepatic veins were evaluated by duplex Doppler ultrasound and computed tomography. The adaptive changes secondary to obstruction were analyzed and their significance was correlated with the clinical findings. Four patients presenting unilateral hepatic vein occlusion had unilateral reversed portal flow. Two of them, with lobar liver atrophy and contralateral compensatory hypertrophy required operation; the other two, with normal appearance of the liver, benefitted from conservative treatment. Two patients with bilateral hepatic vein occlusion, intra-hepatic bypasses, bilateral lobar atrophy and caudate lobe hypertrophy, received operations. Intrahepatic unilateral portal flow reversal compensates for unilateral hepatic outflow obstruction. The combination of complete or subtotal hepatic vein obstruction and atrophy–hypertrophy complex predicates advanced disease despite flow reversal or spontaneous shunt.


*HPB Surgery*, 1995, Vol. 8, pp. 231–236
Reprints available directly from the publisher
Photocopying permitted by license only

© 1995 OPA (Overseas Publishers Association)
Amsterdam B.V. Published in The Netherlands
by Harwood Academic Publishers GmbH
Printed in Malaysia

### Evaluation of Adaptive Changes by Non-Invasive Imaging in Hepatic Vein Outflow Obstruction

P. GERTSCH,* J.-N. VAUTHEY,* C. LOOSER,[†] J. TRILLER,[†] and L. H. BLUMGART*

* Clinic of Visceral and Transplantation Surgery and the [†] Department of Radiology, Inselspital Bern, University of Bern, 3010 Bern – Switzerland

*(Received February 1, 1994)*

Hepatic vein outflow obstruction induces remarkable changes of intra–hepatic blood circulation; the significance of these changes remains uncertain. Six patients with obstruction of the hepatic veins were evaluated by duplex Doppler ultrasound and computed tomography. The adaptive changes secondary to obstruction were analyzed and their significance was correlated with the clinical findings. Four patients presenting unilateral hepatic vein occlusion had unilateral reversed portal flow. Two of them, with lobar liver atrophy and contralateral compensatory hypertrophy required operation; the other two, with normal appearance of the liver, benefitted from conservative treatment. Two patients with bilateral hepatic vein occlusion, intra-hepatic bypasses, bilateral lobar atrophy and caudate lobe hypertrophy, received operations. Intrahepatic unilateral portal flow reversal compensates for unilateral hepatic outflow obstruction. The combination of complete or subtotal hepatic vein obstruction and atrophy–hypertrophy complex predicates advanced disease despite flow reversal or spontaneous shunt.

KEY WORDS: Hepatic vein outflow obstruction duplex doppler ultrasound blood flow portal vein

### INTRODUCTION

Investigations for patients with hepatic vein outflow obstruction have classically included hepatic venography and manometry, inferior vena cavography with pressure measurements in the suprahepatic, retrohepatic and infrahepatic vena cava, and portal vein angiography[1–3]. These studies, which are considered as the gold standard for confirming the diagnosis of the Budd–Chiari syndrome, have provided an indirect evaluation of the extent of hemodynamic changes resulting from hepatic vein outflow obstruction[4,5]. Recently, duplex Doppler ultrasound has allowed direct imaging of the hepatic veins and non–invasive evaluation of hepatic outflow obstruction[6–8]. Computed tomography with intravenous contrast also provides useful data on the alterations in liver perfusion and atrophy–hypertrophy changes of the hepatic parenchyma[9,10].

In this study, six patients with unilateral or bilateral occlusion of the hepatic veins were evaluated by non–invasive imaging studies, with particular attention to the flow patterns in the portal vein and its intra–hepatic branches. The adaptive changes secondary to obstruction were analyzed and their significance was correlated with the clinical findings.

### PATIENTS AND METHODS

Between January 1989 and December 1991, 6 women (age range: 22–45 years) with hepatic vein outflow obstruction were evaluated. Informations on patients' age diagnosis, symptoms, treatment and outcome are presented in Table 1.

Address for correspondence: P. Gertsch, MD, Dept. of Surgery, University of Hong Kong, Queen Mary Hospital, Pokfulam Road, Hong Kong.

**Table 1** Diagnosis and clinical characteristics of the patients

| *Patients* | 1 | 2 | 3 | 4 | 5 | 6 |
|---|---|---|---|---|---|---|
| Age | 45 | 23 | 23 | 35 | 22 | 27 |
| Etiology | Pcy | Pcy | E | Tumor | Pcy | Pcy |
| Duration of symptoms (months) | 6 | 3 | 1 | 3 | 21 | 29 |
| Pain | + | | + | + | | + |
| Fever | | | + | + | | |
| Cachexia | + | + | | | + | + |
| Ascites | + | + | | | + | + |
| Palpable liver | + | + | | + | + | + |
| Treatment | p-c side-to-side | m-c | anti coagulants | | m-c Clatworthy | m-c |
| Outcome | improved | improved | good | stable | dead | improved |

E = estrogen
Pcy = polycythemia vera
p-c = porta-caval shunt
m-c = meso-caval shunt

Symptoms lasted from 1 to 29 months prior to presentation. Patient 3 had a short history of right upper quadrant pain. She underwent cholecystectomy for cholelithiasis and discoloration of the liver was noted during operation. Further investigations including duplex Doppler ultrasound and arteriography revealed multiple thrombosis, including the celiac axis, the hepatic and splenic arteries, the left hepatic vein and the main portal vein. In the absence of detected hematologic disorders, the only risk factor for thrombosis was the intake of oral contraceptives. Patient 4 presented with epigastric pain and temperature before the diagnosis of an unresectable hepatocellular carcinoma. In patient 5, Budd–Chiari syndrome had been present for 21 months and conservative treatment with diuretics had been attempted. In patient 6, symptoms recurred despite prior treatment with retro–hepatic vena cava stenting and atrio–hepatic shunt 20 months earlier[11].

Investigations included abdominal dynamic computed tomography in all cases. Paracentesis prior to investigations was performed in patients with significant ascites.

Ultrasound studies were done with duplex Doppler equipment (Toshiba Sonolayer V SSH–100 A and Acuson 128) in all cases. A sectoral or curved array transducer of 3.5 Mhz was used. Examination was performed in the supine position, after overnight fasting.

The infra–, retro– and supra–hepatic vena cava was investigated in B–mode and the region of the hepatic veins was scanned for identification of the confluence with the inferior vena cava. Presence, direction and characteristics of blood flow were then analyzed. The angle between the longitudinal axis of the vessel and the ultrasonic beam did not exceed $60^0$. Positive duplex Doppler shift was defined as blood flow moving towards the transducer and negative Doppler shift as blood flow moving away from the transducer[12]. Duplex Doppler frequency analysis was also recorded for investigation of flow characteristics. Similarly, the intra– and extra–hepatic branches of the portal vein were identified in B–mode examination before duplex Doppler analysis of flow characteristics was undertaken. Cavography and pressure measurements (hepatic vein free wedged, suprahepatic and infrahepatic vena cava) were performed in patients 1, 2, 5, and 6.

### RESULTS

Computed tomography identified different patterns of lobar atrophy–hypertrophy (Fig. 1). Atrophy of the right and hypertrophy of the left lobe of the liver were the most important findings in cases 1 and 2. A defect corresponding to a necrotic area in the right lobe of the liver was demonstrated in patient 3. A tumor involving segments 2, 3 and 4 of the liver was present in patient 4. Marked hypertrophy of the caudate lobe was observed in cases 5 and 6, associated with atrophy of the right lobe in patient 5 and atrophy of both right and left lobes in patient 6. In patient 5, CT scan demonstrated an intrahepatic vascular channel joining the left branch

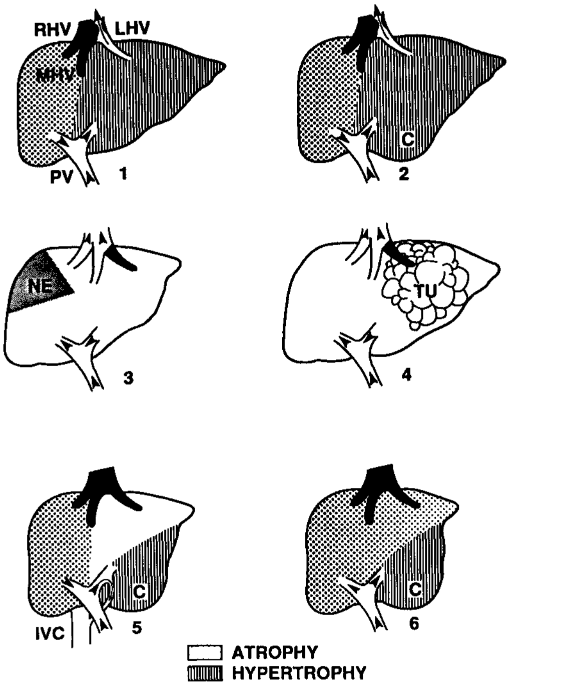

**Figure 1** Schematic illustration of adaptive changes in 6 patients with hepatic outflow obstruction. Direction of hepatic blood flow, extent of hepatic vein obstruction and pattern of liver atrophy-hypertrophy complexes are depicted. RHV = Right Hepatic Vein, LHV = Left Hepatic Vein, MHV = Middle Hepatic Vein, PV = Portal Vein trunk, IVC = Inferior Vena Cava, C = Caudate lobe, NE = necrosis, TU = tumor.

of the portal vein and the retro–hepatic vena cava (porto–caval communication) (Fig. 2).

The anatomy and the direction of flow in the hepatic vessels were analyzed by duplex Doppler ultrasound (Fig. 1). In patients 1 and 2, the right and middle hepatic veins were occluded; the left hepatic vein was patent but narrowed on B mode ultrasound, with high velocities and hepatofugal direction of the blood flow. Blood flow was hepatopetal in the main and left portal vein, and reversed (hepatofugal) in the right portal vein (Fig. 3). In patient 3, extensive vascular thrombosis was observed, with occlusion of the celiac axis, hepatic and splenic arteries. The left hepatic vein also was occluded. Floating clots were present in B mode examination in the main portal vein. The flow was hepatopetal in the main and right portal vein and reversed (hepatofugal) in the left portal vein. Similar hemodynamic findings were observed in patient 4 where tumor compressed and occluded the left hepatic vein. The right, middle and left hepatic veins were occluded in patients 5 and 6. In patient 5, who presented a spontaneous intra–hepatic portacaval shunt, flow direction in the portal vein was hepatopetal. In patient 6, reversed flow was observed in the middle hepatic vein which drained into a patent caudate hepatic vein. The direction of blood flow in the main, right and left branches of the portal vein was hepatopetal.

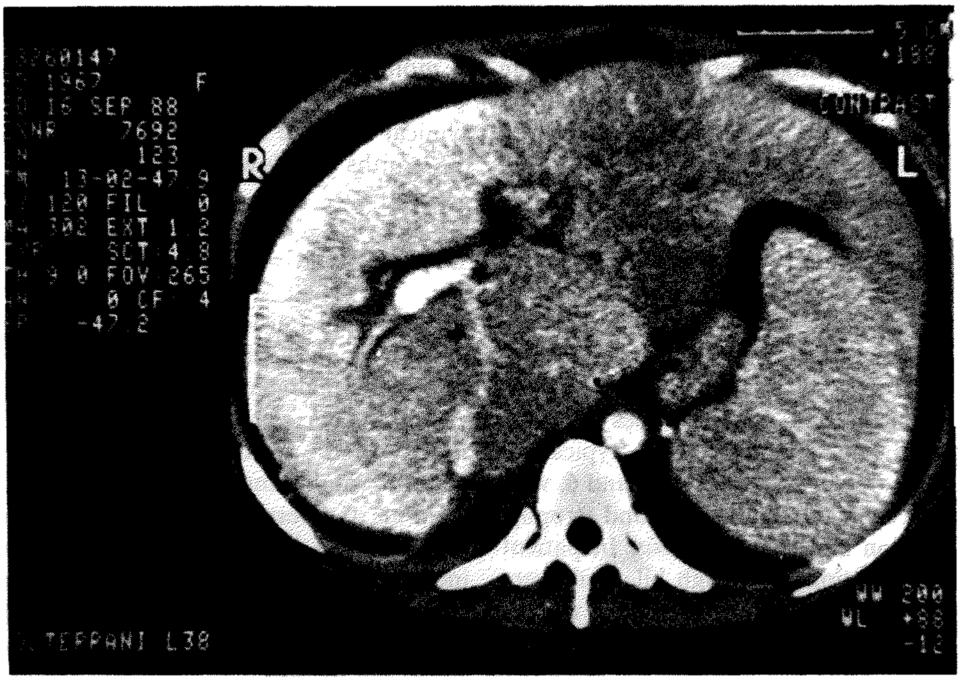

**Figure 2** CT scan demonstrating hypertrophy of the caudate lobe and a vascular channel (->) joining the portal vein to the vena cava (patient 5).

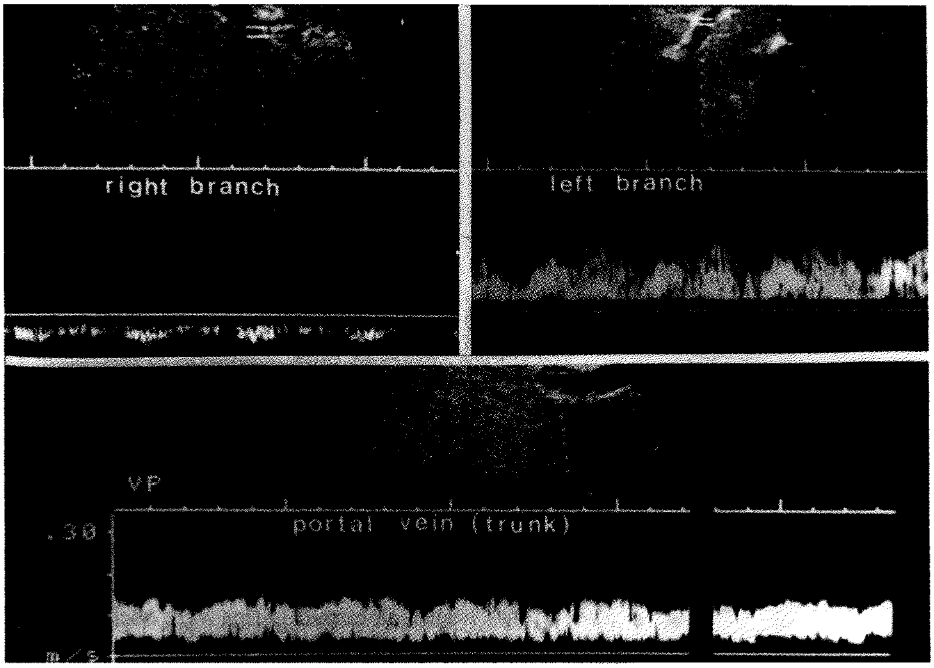

**Figure 3** Duplex Doppler ultrasonography showing hepatopetal flow in the left branch and trunk of the portal vein and hepatofugal flow in the right portal branch (Doppler probe is held in inter-costal position) (patient 2).

Surgical treatment was offered to patients 1, 2, 5 and 6 who had failed to respond to conservative treatment (Table 1). Preoperative cavography and pressure measurements revealed narrowing of the retro–hepatic vena cava. Pressure gradients between the supra–and infra–hepatic vena cava ranged between 4 and 43 mm Hg. Intra–operative portacaval gradients ranged from 8 to 18 mm Hg. Surgical decompression of the liver by portacaval or mesocaval shunt was successful in patients 1, 2 and 6. A retro–hepatic intracaval expandable stent was inserted percutaneously and a side–to–end mesocaval Clatworthy shunt[13] performed in patient 5, who presented a significant stenosis of the retro–hepatic vena cava with a high pressure gradient. This patient died post–operatively of liver insufficiency following shunt thrombosis.

Liver biopsies of the right and left lobes were obtained intraoperatively. Findings were typical of Budd–Chiari syndrome in patients 1, 2, 5 and 6, with a varying extent of centrilobular necrosis and atrophy, sinusoidal congestion and septal fibrosis. In patient 1 and 2, typical alterations, although bilateral, were predominant on the right side; in patient 5, typical alterations were present in the right and in the left lobe, with thrombi demonstrated in centrilobular veins of the caudate lobe; in patient 6, histologic findings were similar on both sides.

The postoperative condition in patients 1, 2 and 6 has been satisfactory, with control of the ascites and proven shunt patency at 4, 5 and 12 months of follow–up. In patient 3, long–term anticoagulant treatment (acenocoumarol) resulted in complete recovery after 9 months of follow–up. Patient 4 was treated with palliative radioembolization and is still alive without clinical evidence of hepatic outflow obstruction after 12 months of follow–up.

### DISCUSSION

Hepatic vein outflow obstruction can be caused by a wide range of disorders including trauma, benign diseases or malignant conditions[14]. The severity of the symptoms correlates with the extent of the obstruction[15]. In the syndrome described by Budd[16] and Chiari[17], abdominal pain, ascites and hepatomegaly combine as a result of severe hepatic venous stasis. If left untreated, the obstruction which is often initially incomplete, almost invariably progresses to hepatic vein occlusion, ultimately resulting in fatal liver failure[1]. Remarkable adaptive changes such as lobar hypertrophy, flow reversal or spontaneous shunt have been described, but their significance remains uncertain[18].

Non–invasive studies have provided direct imaging of the venous obstruction and hepatic parenchymal changes. Dynamic computed tomography can demonstrate hepatomegaly, absent hepatic veins, patchy uptake of contrast by the liver, while the caudate lobe may be preferentially perfused and hypertrophied[9, 10]. Duplex Doppler ultrasound has been used in hepatic outflow obstruction to delineate the anatomy of the obstruction (B–Mode) and to determine hepatic vein outflow characteristics (Doppler)[6–8]. On B–mode, the hepatic veins are typically narrowed, eventually appearing as comma–shape structures in the later stage of the disease. The development of intra–hepatic venous channels diverting the flow from occluded to patent hepatic veins can be demonstrated by duplex Doppler ultrasound (case 6)[8].

In previous reports on duplex Doppler blood flow analysis in Budd–Chiari syndrome, little attention has been given to the changes of intrahepatic portal flow resulting from hepatic venous outflow obstruction. Duplex Doppler analysis shows spontaneous adaptive changes in liver circulation to *unilateral* hepatic vein occlusion. The portal venous system on the affected side becomes an outflow tract, which drains into the portal venous system of the unaffected side (Fig. 1 patients 1–4). Hepatic outflow obstruction results in lobar liver atrophy on the side of complete obstruction and hypertrophy on the incompletely obstructed contralateral side. As venous drainage of the caudate lobe into the retrohepatic vena cava remains patent, some degree of associated caudate lobe hypertrophy is also observed. When obstruction is bilateral, intra–hepatic bypasses develop and shunt blood from obstructed to patent veins (patients 5 and 6).

Reversal of portal venous flow in a territory of hepatic vein occlusion has been interpreted as a possible circulatory compensation more than 50 years ago[19]. Surgical procedures designed to decompress the portal circulation have been used to prevent progression of liver changes associated with hepatic vein obstruction [2,3,20,21]. Experimental and clinical studies have shown that acute lesions resulting from hepatic outflow obstruction can be reversed by side to side portacaval shunt[2]. The natural history of the disease has been altered by surgical decompression and long–term survival achieved[3,22].

In experimental studies up to 55% of the hepatic venous drainage can be impaired without resulting in liver injury[23]. Ligation of one or two hepatic veins without liver resection has been reported to be safe after trauma or during liver surgery[15]. Two patients with portal vein flow reversal had remarkably mild symptoms and tolerated the occlusion of one hepatic vein while adequately decompressing via two patent contralateral hepatic veins (patients 3 and 4). Absence of significant atrophy–hypertrophy complex in these patients is further evidence of the efficacy of this type of spontaneous decompression.

In contrast, 4 patients presenting with abdominal pain, intractable ascites and cachexia (cases 1, 2, 5, 6) required surgical decompression. Neither reversal of flow with a remaining partially patent hepatic vein (patients 1 and 2), nor spontaneous shunts through collateral channels (patients 5 and 6) prevented the progression of the disease. In these four patients, the atrophy–hypertrophy complex observed on computed tomography exemplify the ultimate adaptive changes of liver circulation to severe but incomplete hepatic outflow obstruction. The inefficacy of reversed portal flow in decompressing the liver via a contralateral stenosed hepatic vein is further confirmed by the presence of bilateral histologic alterations characteristic of hepatic venous stasis. Computed tomography and duplex Doppler ultrasound alterations provide a detailed analysis of spontaneous hemodynamic and hepatic adaptive changes secondary to hepatic venous outflow obstruction. The extent of these changes correlates with the severity of the disease. Careful analysis of blood flow direction in the intrahepatic branches of the portal vein may provide useful information for ultrasound diagnosis of Budd–Chiari syndrome.

### REFERENCES

1. Tavill, A. S., Wood, E. J., Kreel, L, Jones, E. A., Gregory, M. and Sherlock, S. (1975) The Budd–Chiari syndrome: correlation between hepatic scintigraphy and the clinical, radiological and pathological findings in nineteen cases of hepatic venous outflow obstruction. *Gastroentero.*, **68**, 509–18.
2. Orloff, M. J. and Johansen, K. H. (1978) Treatment of Budd–Chiari syndrome by side–to–side portacaval shunt: experimental and clinical results. *Ann. Surg.*, **188**, 494–512.
3. Orloff, M. J., Orloff, M. S. and Daily, P. O. (1992) Long-term results of treatment of Budd–Chiari syndrome with portal decompression. *Arch. Surg.*, **127**, 1182–1188.
4. Brink, A. J. and Botha, D. (1955) Budd–Chiari syndrome: diagnosis by hepatic venography. *Brit. J. Rad.*, **28**, 330–331.
5. Deutsch, V., Rosenthal, T., Adar, R. and Mozes M. (1972) Budd–Chiari syndrome. Study of angiographic findings and remarks on etiology. *Am. J. Roentgen.*, **116**, 430–439.
6. Grant, E. G., Perela, R., Tessler, F. N., Lois, J. and Busuttil R. (1989) Budd–Chiari syndrome: the results of duplex and color Doppler imaging. *Am. J. Roentgen.*, **152**, 377–381.
7. Hosoki, T., Kuroda, C., Tokunaga, K., Marukawa, T., Masuike M. and Kozuka, T. (1989) Hepatic venous outflow obstruction: evaluation with pulsed duplex sonography. *Radiology*, **170**, 733–737.
8. Bolondi, L., Gaiani, S., Li Bassi, S., Zironi, G., Bonino, F., Brunetto, M. and Barbara, L. (1991) Diagnosis of Budd–Chiari syndrome by pulsed Doppler ultrasound. *Gastroentero.*, **100**, 1324–1331.

9. Rossi, P., Sposito, M., Simonetti, G., Sposato, S. and Cusumano, G. (1981) CT diagnosis of Budd–Chiari syndrome. *J. Comp. Assist. Tomog.*, **5,** 366–369.
10. Harter, L. P., Gross, B. H., St. Hilaire, J., Filly, R. A. and Goldberg, H. I. (1982) CT and sonographic appearance of hepatic vein obstruction. *Am. J. Roentgen.*, **139,** 176–178.
11. Banski, G., Ernest, C., Jenni, R., Zollikofer, C., Burger, H.R. and Senning, A. (1986) Treatment of Budd–Chiari syndrome by dorsocranial liver resection and direct hepatoatrial anastomosis. *J. Hepat.*, **2,** 101–112.
12. Wells, PNT. (1988) Instrumentation including color flow mapping. In Clinical application of Doppler ultrasound, edited by K. J. W. Taylow, P. N. Burns, P. N. T. Wells, pp 26–45. New York: Raven Press.
13. Clatworthy, H. W. Jr., Wall, T. and Watman, R N. (1955) A new type of portal–to–systemic venous shunt for portal hypertension. *Arch. Surgery*, **71,** 588–596.
14. Kelsey, M. P. and Comfort, M. W. (1945) Occlusion of the hepatic veins: a review of twenty cases. *Arch. Int. Med.*, **75,** 175–183.
15. Ou, Q. J. and Hermann, R. E. (1984) The role of hepatic veins in liver operations. *Surg.*, **95,** 381–391.
16. Budd, G. (1845) On diseases of the liver. First Edition, p 146 London: J & A Churchill Ltd.
17. Chiari, H. (1899) Uber die selbständige Phlebitis obliterans der Hauptstämme der Venae hepaticae als Todesursache. Beitrag für Pathologische Anatomie, **26,** 1–17.
18. Pollard, J. J. and Nebesar, R. A. (1967) Altered hemodynamics in the Budd–Chiari syndrome demonstrated by selective hepatic and selective splenic angiography. *Radiology*, **89,** 236–243.
19. Altschule, M. D. and White, C. (1939) Chiari's syndrome in a patient with polycythemia vera. *N. Eng. J. Med.*, **220,** 1320–1325.
20. Orloff, M. J., Daily, P. O. and Girard, B. (1992) Treatment of Budd–Chiari syndrome due to inferior vena cava occlusion by combined portal and vena caval decompression. *Am. J. Surg.*, **163,** 137–143.
21. Vons, C., Bourstyn, E., Bonnet, P., Smadja, C., Szekely, A.M. and Franco, D. (1986) Results of portal systemic shunts in Budd–Chiari syndrome. *Ann. Surg.*, **203,** 366–370.
22. Bismuth, H. and Sherlock, D. J. (1991) Portasystemic shunting versus liver transplantation for the Budd–Chiari syndrome. *Ann. Surg.*, **214,** 581–589.
23. Widmann, W. D., Hales, M.R. and Greenspan, R.H. (1962) The effects of hepatic occlusions. *Am. J. Path.*, **41,** 439–454.

